# High *CHI3L1* expression is associated with glioma patient survival

**DOI:** 10.1186/s13000-016-0492-4

**Published:** 2016-04-27

**Authors:** Giedrius Steponaitis, Daina Skiriutė, Arunas Kazlauskas, Ieva Golubickaitė, Rytis Stakaitis, Arimantas Tamašauskas, Paulina Vaitkienė

**Affiliations:** Laboratory of Neurooncology and Genetics, Neuroscience Institute, Medical Academy, Lithuanian University of Health Sciences, Eiveniu str. 4, Kaunas, LT-50009 Lithuania

**Keywords:** CHI3L1, Chitinase 3-like 1, YKL-40, Glioma, Astrocytoma, Survival, mRNA

## Abstract

**Background:**

Survival of glioma patients with the same tumor histology and grade can vary significantly, and some low-grade gliomas transform to a more malignant phenotype. There is a need of molecular signatures, which are better predictors of the patient diagnosis, outcome of treatment, and prognosis than the diagnosis provided by histopathology. We propose *CHI3L1* mRNA expression as a prognostic biomarker for patients with glioma.

**Methods:**

We measured *CHI3L1* expression with quantitative real time-polymerase chain reaction (qRT-PCR) in the cohort of 98 patients with different grade glioma: 10 grade I pylocytic astrocytomas, 30 grade II diffuse astrocytomas, 20 grade III anaplastic astrocytomas, and 38 grade IV astrocytomas (glioblastomas). Statistical analyses were conducted to investigate the association between *CHI3L1* mRNA expression levels and patient clinical variables.

**Results:**

We demonstrated that mRNA expression of *CHI3L1* was evidently higher in glioblastoma than in lower grade glioma tissues. We evaluated correlations between *CHI3L1* expression, clinicopathological characteristics, and the outcomes of the patients. Patients with high *CHI3L1* expression had a shorter overall survival (*p* < 0.001).

**Conclusions:**

Findings presented in our study showed that increased mRNA level of *CHI3L1* could be associated with the progression of astrocytoma and poor patient survival not only for glioblastoma, but for lower grade astrocytoma tumors as well. Further investigation will be required to evaluate *CHI3L1* value as a molecular marker for astrocytoma prognoses and for novel treatment strategies against all grade astrocytomas.

## Background

Brain tumours are classified according to the WHO classification of CNS tumours (2007 which is based on histological characteristics of the tumour) [1]. The previous works demonstrated that molecular signatures allow a better characterisation of the pathology than the current clinical scheme based on histopathological classification [2, 3]. According to the WHO classification, gliomas are subdivided into the (rare) ependymomas, oligodendrogliomas, and astrocytomas, which are the largest group of gliomas [4]. Astrocytoma has a well differentiated variant, known as pilocytic astrocytoma (WHO grade I, diffuse astrocytoma (WHO grade II), anaplastic astrocytoma (WHO grade III) and astrocytoma grade IV, which is known as glioblastoma multiforme. Glioblastoma is one of the most malignant forms of human brain tumour. Glioblastoma is clinically classified as primary or secondary subtypes depending on whether it was diagnosed as a de novo tumor or it derived from gliomas of lower grade, respectively [1]. However, survival of glioma patients with the same tumor histology and grade can vary significantly, and some low-grade gliomas transform to a more malignant phenotype [5]. There is need of molecular signatures, which are a better predictor of the patient diagnosis, outcome of treatment, and prognosis than the diagnosis provided by histopathology.

Chitinase 3-like 1 (CHI3L1) plays a role in cell proliferation, differentiation, apoptosis, angiogenesis, inflammation and extracellular tissue remodeling [6]. CHI3L1 is located on chromosome 1q32.1, and the product, YKL-40, a 40-kDa glycoprotein, is secreted by numerous human cells such as cartilage, synovium, endothelial cells, inflammatory cells, and cancer cells [7]. There are data that, presence of YKL-40 protein in serum, could be a prognostic predictor of glioblastoma [5, 8]. The high serum levels of the glycoprotein are associated with poor prognosis of various medical, inflammatory and tumour processes [9–12]. These medical, inflammatory, and malignant diseases all possibly contribute to the levels of serum YKL-40. In our study, we used real-time quantitative PCR (qRT-PCR) to measure the quantitative expression of CHI3L1 in different grade astrocytoma tissues without the influence of other malignancies or medical diseases.

## Methods

### Patients and tissue samples

In total 98 post-operative samples obtained from patients diagnosed with different malignancy grade gliomas were analyzed: 10 grade I pylocytic astrocytomas, 30 grade II diffuse astrocytomas, 20 grade III anaplastic astrocytomas, and 38 grade IV astrocytomas (glioblastomas). All glioma tumor samples were collected in Neurosurgery Clinics of Hospital (NCH) of Lithuanian University of Health Sciences (Kaunas, Lithuania) during the period from the year 2003 to 2014 with informed consent from patients. Tumor samples were collected, following written informed consent, in accordance with the Lithuanian regulations and the Helsinki Declaration. Written informed consent was obtained for every patient under the approval of the Ethics Committee, Lithuanian University of Health Sciences. Database closure was in March 2015. Diagnoses were established by pathologists at the NCH according to the World Health Organization (WHO) classification. Glioma samples were stored in liquid nitrogen until analysis. The following clinical data were collected for each patient: age at the time of the operation, gender, and patient status. The overall survival of the patient was calculated from the date of the operation to the date of death or the last recorded contact with the live patient (censored). None of the patients had received chemotherapy or radiation before surgery.

### Methylation specific PCR

Brain tumor tissue specimens after dissection were snap-frozen in liquid nitrogen and stored until analysis. Tumor DNA was purified from 50–100 mg of frozen tissue using ZR Genomic DNA Tissue MiniPrep (Zymo Research) according to manufacturer’s protocol. The concentration of DNA was measured using NanoDrop 2000 Spectrophotometer (Thermo Scientific) before DNA bisulfite-treatment. Obtained values were used in subsequent phases of the study as approximate quantity. Methylation status of *CHI3L1* gene promoter was determined by bisulfite treatment of DNA. 400 ng of total genomic DNA was modified using EZ DNA Methylation Kit (Zymo Research). Bisulfite-treated DNA was eluted in 40 μl nuclease-free water, and stored in −80 °C until analysis. “Human brain DNA” (Zymo Research, Cat. No. D5018) served as a normal brain tissue control. For negative methylation control normal human blood lymphocyte DNA treated with bisulfite was used. Bisulfite Converted Universal Methylated Human DNA Standard (Zymo Research) was used as a positive control for DNA methylation. Promoter methylation was detected by methylation-specific PCR (MSP). Each MSP reaction incorporated approximately 20 ng of bisulfite-treated DNA as template. Specific primers for methylated and unmethylated target DNA sequence were designed using free access online software’s (1–3). In total two primer sets for different CpG dinucleotides sites were used for the study. MSP primers for methylated *CHI3L1* were:

1^st^: 5′- TTTTTATAAAAGGGTTGGTTTGTC -3′ (sense)

5′- TAACCCAAATACCTATTTAAAACGC-3′ (antisense)

2^nd^: 5′- TGTTAGATGTTCGTGTAGTCGTTTC-3′ (sense)

5′- CCAAAAATACTTTAAACCCCGAT-3′ (antisense)

and for unmethylated sequence:

1^st^: 5′- TTTTTATAAAAGGGTTGGTTTGTTG-3′ (sense)

5′- AACCCAAATACCTATTTAAAACACC -3′ (antisense)

2^nd^: 5′- TTAGATGTTTGTGTAGTTGTTTTGT -3′ (sense)

5′- CCAAAAATACTTTAAACCCCAAT -3′ (antisense)

Reaction was performed in 15 μl of total volume by using 7.5 μl Maxima Hot Start PCR Master Mix (Thermofisher Scientific) with Hot start Taq DNA polymerase and 10 pmol of each primer (Metabion International AG). MSP was performed for 36 cycles with the reaction starting at 95 °C for 15 s., annealing of 58 °C and 61 °C (1^st^ and 2^nd^ primer pair respectively) for 30 s., and extension of 72 °C for 20 s. Amplification products were analyzed on 2 % agarose gels with ethidium bromide (final conc. 0.05 μg/ml) and documented under UV gel imaging system (Gel Doc XR+ System, BioRad). The presence of a PCR product of the correct molecular weight indicated the presence of either unmethylated or methylated alleles. In case of appearance of both unmethylated and methylated signals, case was considered as being methylated.

### RNA extraction, cDNA synthesis and quantitative RT-PCR

Total RNA from cryogenically homogenized tumor tissue was purified using TRIzol Reagent (Ambion, Life Technologies). To increase the yield of RNA, homogenate was additionally sonicated using ultrasound (500-W ultrasonic processor, Cole Parmer). The concentration of RNA was measured using NanoDrop 2000 Spectrophotometer (Thermo Scientific). Obtained values were used in subsequent phases of the mRNA quantitation as approximate quantity. Reverse transcription (RT) was carried out using RevertAid H Minus M-MuLV Reverse Transcriptase (Thermofisher Scientific) and random hexamer primers (Thermofisher Scientific) in a total reaction volume of 20 μl according to the manufacturer’s protocol. For inhibition of *m*RNA degradation RiboLock RNase inhibitor (ThermoFisher Scientific) was used. After synthesis cDNA stock was stored at −80 °C. *CHI3L1 m*RNA expression was analyzed using quantitative real-time RT-PCR TaqMan probe assay in 3 replicates on 7500 Fast Real-time PCR detection system (Applied Biosystems) and Relative Quantitation method (ΔCT). Reactions have been assembled into a total volume of 12 μl of each, which included: 15 ng of the cDNA, 6 μl of TaqMan Universal Master Mix II, no UNG (Applied biosystems) with AmpliTaq Gold® DNA Polymerase and Taqman expression probe for *CHI3L1* (assay no: Hs00609691_m1) and nuclease-free water. *β-actin m*RNA expression was analyzed using quantitative real-time RT-PCR SYBR Green I assay on the same detection system. 12 μl of reaction mix consist of 15 ng of the cDNA, 6 μl Maxima Hot Start PCR Master Mix (Thermofisher Scientific) with Hot start Taq DNA polymerase, primers for *β-actin* (5′-AGAGCTACGAGCTGCCTGAC-3′ (sense) and 5′-AGCACTGTGTTGGCGTACAG-3′ (antisense), amplicon length: 184 bp to a total concentration of 0.1 μM, and nuclease-free water. PCR has been carried out for 40 cycles consisting of 95 °C for 30 s., 60 °C for 30 s., and 72 °C for 30 s. Fluorescent data were converted to threshold cycle (CT) measurements. ΔCT values were calculated from averaged replicates CT according to the formula ΔCT = CT *CHI3L1* – CT *β-actin*. Differences between the plates were equalized using reference sample (Human normal brain) Ct values realignment between experiments. To be able to quantify samples in 95 % of cases, samples with standard deviation more than 0.25 (Ct between replicates) were eliminated from analysis. The final result was given as Log2 of 2^–(ΔCT)^ calculation. Human normal brain RNA sample “FirstChoice Human Brain Reference RNA” (Ambion), which was a pool of RNAs assembled from multiple donors from several brain regions, as described by the manufacturer, served as a control sample for standard curve design for *β-actin*.

### Statistical analysis

SPSS Statistics 22 (SPSS Inc., Chicago, IL) software package was used for statistical analysis. Association between *CHI3L1 m*RNA level data and clinical features of glioma patients were analyzed by Chi-Square Test. The Kaplan-Meier method was used to estimate survival functions. For comparing survival time distribution between groups the log-rank test was used. Prognostic factors such as gender, age, pathological grade, CHI3L1 promoter methylation and mRNA expression were first examined individually (univariate analysis), and all factors that had strong impact on survival (*p* < 0.05) were then evaluated jointly in Cox regression analysis (multivariate analysis). Differences in *CHI3L1 m*RNA expression between different glioma malignancy grades were evaluated using the One-way ANOVA analysis. The level of significance was set to *p* < 0.05.

## Results

### Analyses of CHI3L1 expression in different grade gliomas

**Fig. 1:**
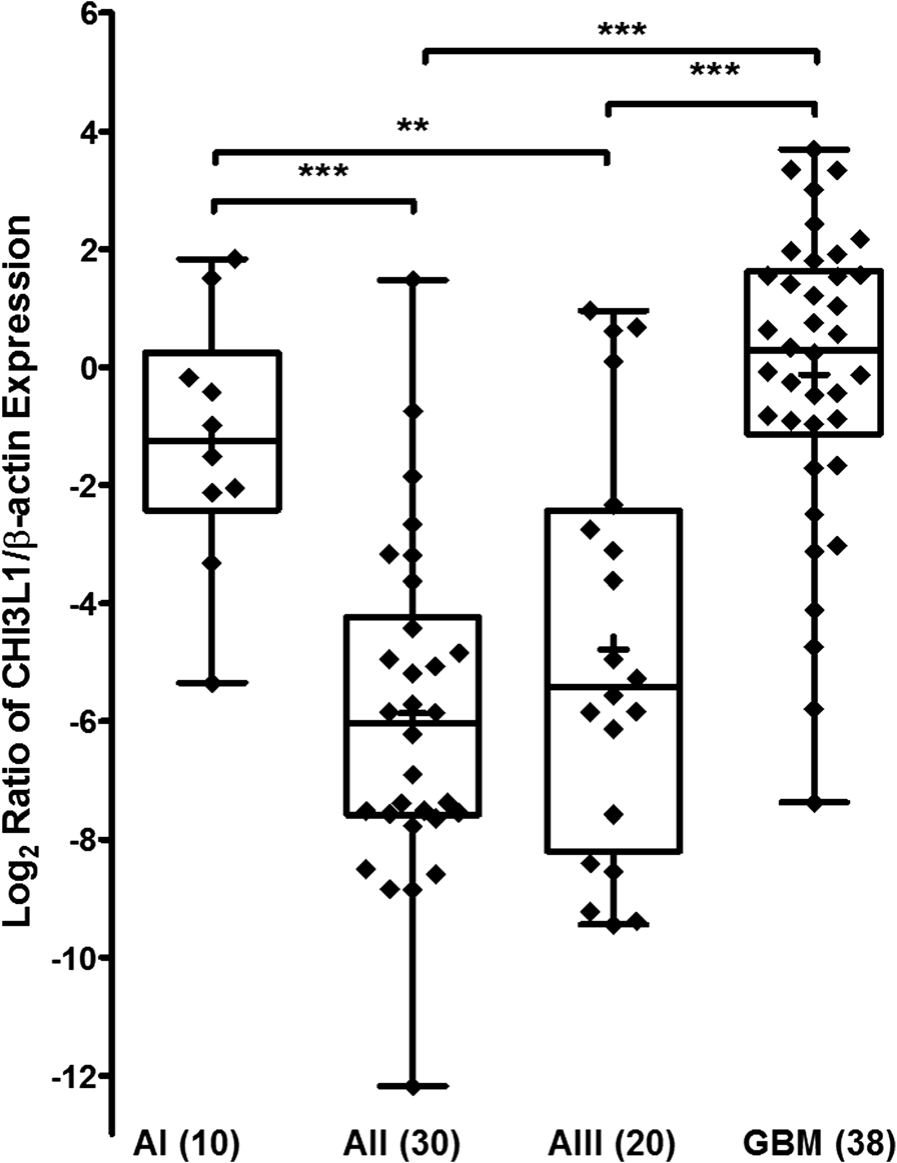
*m*RNA level of *CHI3L1* in different malignancy grade of astrocytic gliomas. Data expressed as log2 of fold change in CHI3L1/β-actin *m*RNA expression. AI–AIII - grade I-III astrocytoma’s; GBM – glioblastoma. Horizontal lines mark median, while plus indicator (+) shows mean of *CHI3L1* expression in the group. Significant decrease of *CHI3L1 m*RNA was observed in grade II and III astrocytoma’s compared to GBM (*p* < 0.001) and I grade glioma (respectively *p* < 0.001 and *p* = 0.032)

At first we checked whether the *CHI3L1 m*RNA level is associated with the different histopathological grade of glioma. One-way ANOVA analysis showed significant *m*RNA level differences among tumor malignancy groups (F = 27.85; *p* < 0.001). To asses which groups significantly differ Pos-Hoc Multiple comparison Hochberg’s GT2 test was applied. Multiple comparison analysis showed significantly higher *CHI3L1 m*RNA expression in glioblastoma (grade IV) cohort compared with II and III grade gliomas (*p* < 0.001). Besides that, *CHI3L1 m*RNA expression in glioma grade I also showed to be significant higher than compared with grade II and III, respectively *p* < 0.001 and *p* = 0.032. No important *m*RNA level difference between gliomas grades I and IV and grades II and III were observed (*p* > 0.05) (Fig. 1).

**Fig. 2:**
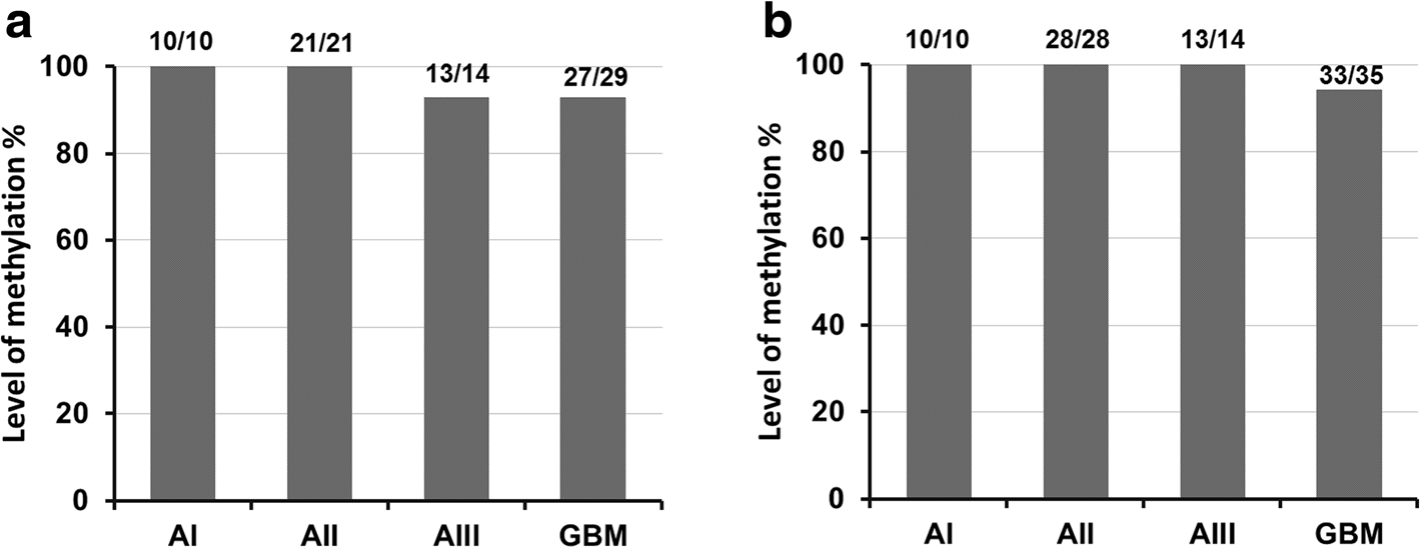
Methylation analysis of *CHI3L1* in different grade gliomas. **a** Methylation frequency analyzed with first primer set while (**b**) with second primer set. Both primer sets detects separate CpG dinucleotides in *CHI3L1* promoter CpG island. No significant association was observed among malignancy grades and *CHI3L1* methylation frequency analyzed with 1-st primer set (*χ*2 = 3,815, df = 3, *p* = 0.282) and 2-nd primer set (*χ*2 = 1,646, df = 3, *p* = 0.649)

Next we decided to examine whether the increase of *CHI3L1 m*RNA level in glioblastomas is due to the promoter methylation of the *CHI3L1* gene. For this purpose, methylation status of 2 sites of *CHI3L1*gene promoter was analyzed by MS-PCR followed by DNA bisulfite treatment. *CHI3L1* methylation status showed no link between tumor malignancy grade (Chi-square test Likelihood Ratio *p* > 0.05) or gene *m*RNA level (Mann–Whitney test *p* > 0.05) on both analyzed promoter sites (see Fig. 2).

### Correlation of CHI3L1 with clinicopathological characteristics and patient survival

To determine the significance of the increased *CHI3L1 m*RNA expression in glioma, we analyzed the relationship between *CHI3L1 m*RNA levels and the clinicopathological features of glioma tumor patients. For this purpose, *CHI3L1 m*RNA level values obtained from the complete set of 98 different grade glioma samples have been divided into three categories: values that were lower than or equal to the 25th percentile were ranked as “low” *CHI3L1 m*RNA level, values falling between the 25th and 75th percentile range were considered as “medium” *CHI3L1 m*RNA level, and values that were higher than or equal to the 75th percentile were ranked as “high” *CHI3L1 mRNA* levels. According to the RT-PCR results, “low” *m*RNA levels of *CHI3L1* were determined for 24 (24.5 %), “medium” *CHI3L1* levels were determined for 50 (51 %), and “high” *CHI3L1* levels were determined for 24 (24.5 %) glioma patients. As shown in Table 1, we found positive significant relationship between *CHI3L1 mRNA* level and high tumor malignancy (*p* < 0.001). This suggested that *CHI3L1* is positively associated with glioma progression. *CHI3L1 m*RNA levels were also associated with patient age (*p* < <0.001), but gender (p >0.05, Table 1).

**Table 1:** Association of CHI3L1 *m*RNA expression in human glioma tissues with different clinicopathological features

Variable	Number of cases	*CHI3L1 m*RNA level			*p*-value
		Low *n* (%)	Medium *n* (%)	High *n* (%)	
Overall	98	24 (24.5)	50 (51)	24 (24.5)	
Age (Years)					<0.001
≤50	53	23 (43.4)	24 (45.3)	6 (11.3)	
>50	45	1 (2.2)	26 (57.8)	18 (40.0)	
Gender					0.683
Male	43	12 (27.9)	22 (51.2)	9 (20.9)	
Female	55	12 (21.8)	28 (50.9)	15 (27.3)	
Pathological grade (WHO)					<0.001
Grade I	10	0 (0)	8 (80)	2 (20)	
Grade II	30	16 (53.3)	13 (43.3)	1 (3.4)	
Grade III	20	7 (35.0)	10 (50.0)	3 (15.0)	
Grade IV	38	1 (2.6)	19 (50.0)	18 (47.4)	

**Fig. 3:**
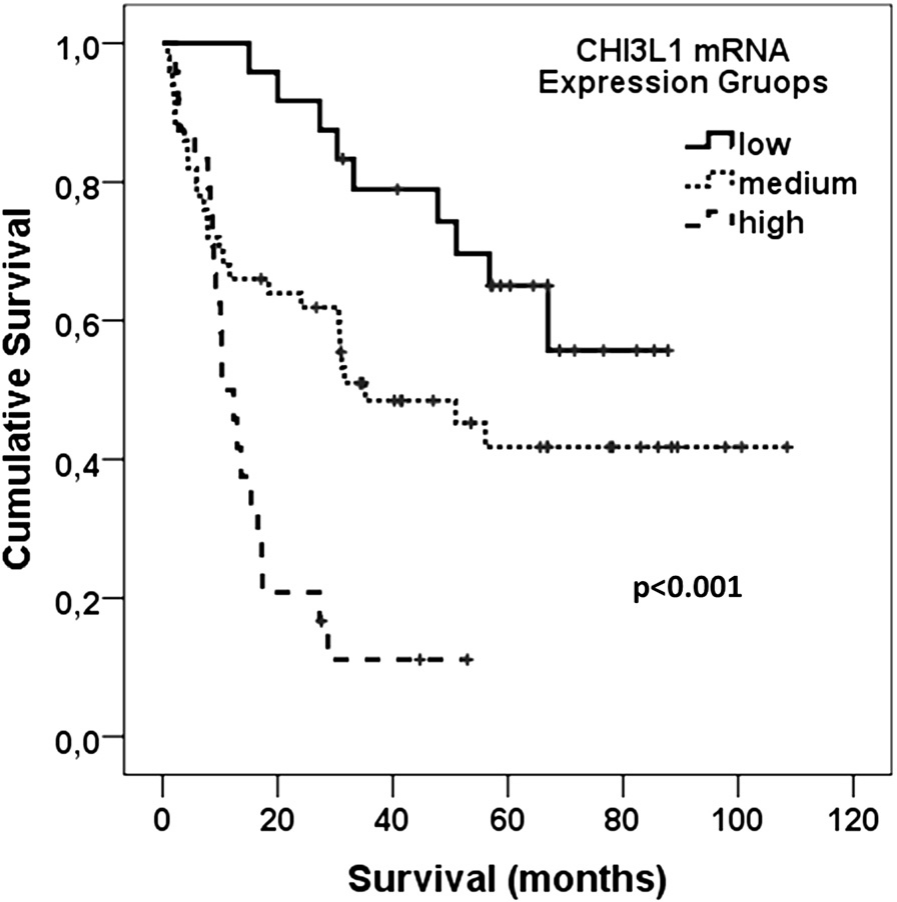
Kaplan-Meier survival curves show strong secession of patient overall survival in high medium and low *CHI3L1 m*RNA expression groups (Log-rank test, *χ*2 = 25.174, df = 2, *p* < 0.001) thus suggesting *CHI3L1* impact for gliomagenesis

**Fig. 4:**
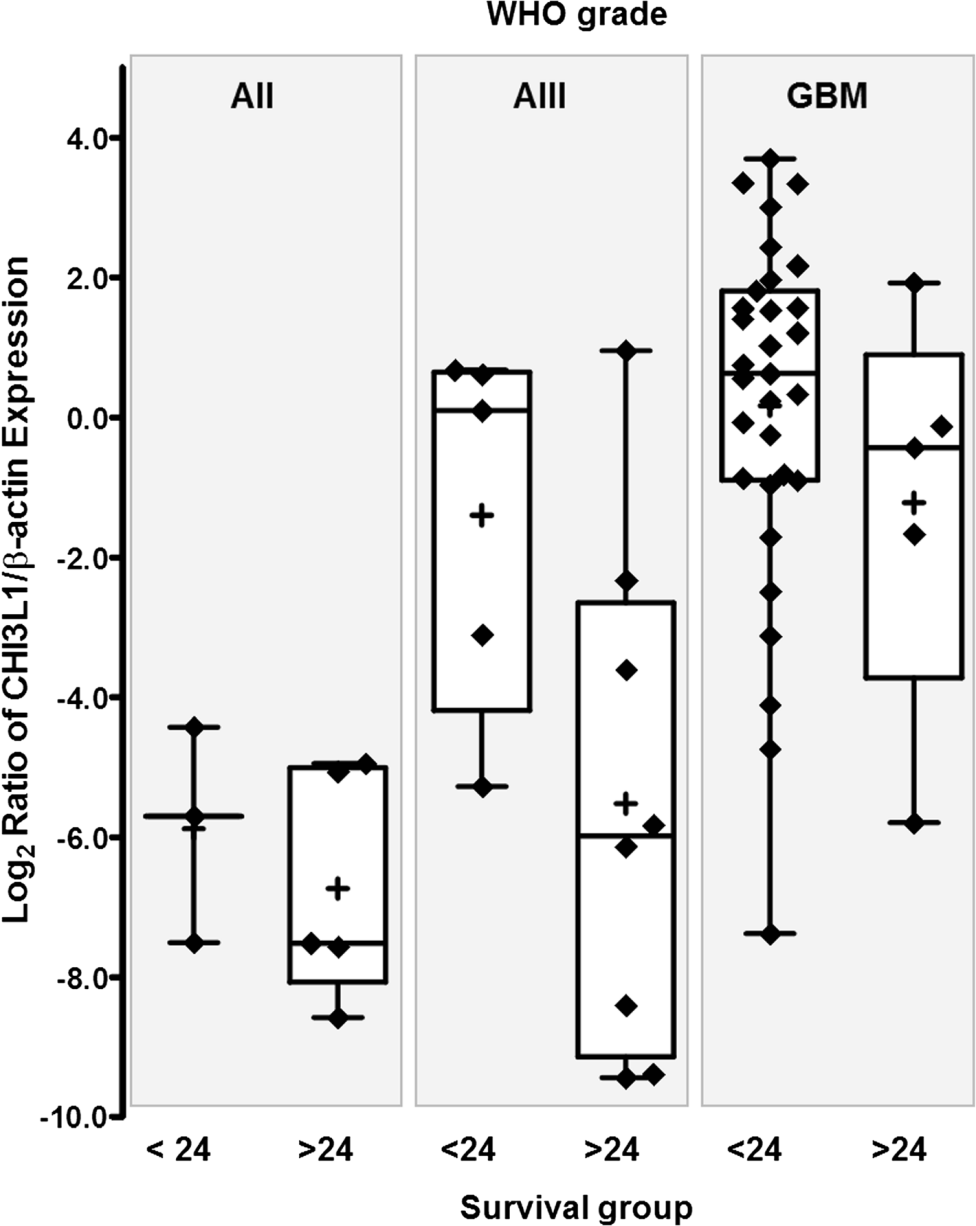
Association between *CHI3L1* mRNA expression and patient survival. The line inside boxes represent median, the plus symbol (+) represent the mean and the lower and upper edges of the boxes represent 25^th^ and 75^th^ percentiles, respectively. Only dead cases were included in the analysis and this has been evidently limiting factor to determine statistically significant difference between CHI3L1 expression groups in patient survival groups (*t*-test *p* > 0.05)

The link between *CHI3L1* expression and glioma pathological grade indicated us to check the association between patient survival and Chitinase 3-Like 1 expression. The Kaplan-Meier analysis using the log-rank test showed strong association between patient overall survival and *CHI3L1 m*RNA expression (Log-rank test, *χ*2 = 25.174, df = 2, *p* < 0.001) (see Fig. 3). Glioma patient with low *CHI3L1* mRNA expression had significantly higher chance of longer survival when compared with medium and especially high *CHI3L1* expression. It should be noted that these data are in the line with gene mRNA expression distribution across tumor grade – higher CHI3L1 expression was intrinsic for glioblastoma tissue. Relation between patient overall survival and *CHI3L1 m*RNA expression prompt us to check whether *CHI3L1 m*RNA level differ in WHO grade and patient survival groups. *CHI3L1 m*RNA expression data was divided into two groups according to patient survival <24 and >24 months (after removal of tumor) in each WHO grade. Astrocytoma grade I tumors were omitted because all grade I cases were alive on the time of analysis. Only dead subjects were included in the analysis. No statistically significant connection but tendency between *CHI3L1* expression and patient survival was observed in all analysed WHO groups most likely due to small number of cases (*t*-test: AII *p* = 0.493; AIII *p* = 0.054; GBM *p* = 0.27) (see Fig. 4).

To assess the prognostic potential for independent variables (such as *CHI3L1* promoter methylation, mRNA expression, astrocytoma pathological grade, age and gender) associated with patient survival univariate Cox analysis was performed. Astrocytoma grade I samples were retracted from the analysis due to patient status (all the patients were alive on the time of analysis). Univariate Cox analysis showed that *CHI3L1* mRNA expression (*p* < 0.001), astrocytoma pathological grade (*p* < 0.001) and patient age (*p* < 0.001) had a significant association as independent variables with the overall survival of the patients, while *CHI3L1* promoter methylation status and patient gender had no significant connection (*p* > 0.05). All factors that had strong impact on survival were then evaluated jointly in multivariate Cox analysis. Astrocytoma grade II was selected as reference group when analyzing qualitative data. Multivariate analysis confirmed that astrocytoma grade, *CHI3L1* mRNA expression level and patient age were considered as independent prognostic factors (see Table 2). As expected Cox analysis revealed that astrocytoma pathological grade had strong impact for patient survival as independent factor. Hazard ratio (HR) of shorter survival for grade III patient’s was 2.95, *p* = 0.017 while for GBM almost twice higher – 5.34, *p* = 0.002 when compared to grade II astrocytoma. The second most important independent factor which was shown by multivariate cox analysis is the increment of *CHI3L1* mRNA expression. Higher *CHI3L1* mRNA level increases the risk of shorter survival for patient, HR =1.119, *p* = 0.038. The age of patients was the last appointed independent factor which slightly increases the risk of shorter survival for older patient, HR =1.033, *p* = 0.022 (see Table 2).

**Table 2:** Multivariate Cox regression analysis showed that *CHI3L1* mRNA level is one of the three analyzed independent variables influencing the survival of the patients

Factor	HR 95 % CI	B	*p*-value
Astrocytoma grade (WHO)			0.007
Grade III	2.949 (1.213–7.17)	1.082	0.017
Grade IV (GBM)	5.342 (1.814–15.73)	1.676	0.002
CHI3L1 mRNA	1.119 (1.006–1.244)	0.112	0.038
Age	1.033 (1.005–1.061)	0.032	0.022

## Discussion

One of the most dramatically induced genes in GBM is *CHI3L1* [13, 14]. A wealth of clinical evidence has also revealed that elevated serum levels of *CHI3L1* in GBM are positively correlated with cancer invasiveness, radioresistance, recurrence, and reduced patient survival times [15]. There are data that, *CHI3L1* expression could be a prognostic predictor of glioblastoma [5, 8, 16], although other studies have not supported this role [17]. Pelloski with colleagues used subjective score (0, 1+, 2+) to evaluate CHI3L1 expression and found positive correlation between CHI3L1 staining and short survival [18]. Later the same research group failed to find this correlation, but found that combined CHI3L1/EGFRvIII negative status was associated with better prognosis [19]. In other studies was found no correlation between survival and CHI3L1 mRNA level [16, 17]. Also no correlation was found between IHC staining for CHI3L1 and patient survival [20]. Also, the literature up to date lacks crucial documentation of *CHI3L1* expression with respect to tumor grade and interface with survival. Recently, a number of gene expression array studies have identified *CHI3L1* to be one of the most overexpressed genes in glioblastoma when compared to low-grade glioma and normal brain [13, 14, 21, 22], but most of them were carried out with small sample number. Either several medical and inflammatory diseases have been associated with elevated serum levels of YKL-40, including polycystic ovarian sindrome [23], rheumatoid arthritis [24], diabetes mellitus [25]. These medical, inflammatory, and malignant diseases all possibly contribute to the levels of serum CHI3L1. In our study, we used real-time quantitative PCR (qRT-PCR) to measure the quantitative expression of *CHI3L1* in different grade astrocytoma tissues without the influence of other malignancies or medical diseases. Our data showed that *m*RNA expression level of *CHI3L1* in glioma specimen was associated with tumour malignancy and patient overall survival. Higher mRNA level was more frequent in glioblastoma tissue as compared to grade II and III glioma. Grade I glioma also showed significantly higher *CHI3L1 m*RNA expression as compared to grade II and III glioma, but less than GBM. It is important to mention that grade I glioma expression profile was more similar to healthy brain (RHB) sample and this could indicate that expression of *CHI3L1* is at very beginning stage of alteration in grade I glioma. This suggests that *CHI3L1* expression shifts through gliomagenesis and is downregulated at grade II and III but upregulated in GBM. Next it would be useful to find out what molecular mechanisms are responsible for this shifting, as far as this could be very important for tumour malignancy progression. The *CHI3L1* importance for gliomagenesis was showed by survival analysis. Despite grade I glioma specimen showed similar *CHI3L1* expression profile to glioblastoma specimen Kaplan-Meier curves strongly separate patient with different expression level to diverse survival groups. This demonstrates that *CHI3L1* mRNA expression level could be informative prognostic marker for glioma patient overall survival. Castells and colleagues reported that the expression values from only four transcripts (*CHI3L1*, *LDHA*, *LGALS1*, and *IGFBP3*) were able to distinguish two survival groups in Glioblastoma [26]. Our findings propose that mRNA expression values of *CHI3L1*, could be useful, not only for glioblastoma, but for lower grade astrocytoma diagnosis and prognosis too. Unlike *CHI3L1* mRNA data, promoter methylation status analysis did not reveal significant relevance on gliomagenesis. Such data could be clarified in two theories: wrong selection of gene promoter sequence to analyse; or different gene regulation mechanisms than promoter methylation is intrinsic for *CHI3L1*. Recent discoveries found that CHI3L1 acts on glioblastoma-stem like cells (GSCs) to drive the formation of tumour vascularization and targeting CHI3L1 may compliment conventional anti-angiogenic therapies to provide a substantial clinical benefit to patients with GBM [15].

## Conclusions

Findings presented in our study showed that the increased mRNA level of *CHI3L1* could be associated with the progression of astrocytoma and with poor patient survival not only in the glioblastoma but in the lower grade astrocytoma tumors as well. Further investigation will be required to evaluate *CHI3L1* as a molecular marker for astrocytoma prognoses and for novel treatment strategies against all grade astrocytomas.

### Ethical standards

Experiments described in the manuscript comply with the current laws of the country in which they were performed.
